# Simulation of *E. coli* Gene Regulation including Overlapping Cell Cycles, Growth, Division, Time Delays and Noise

**DOI:** 10.1371/journal.pone.0062380

**Published:** 2013-04-26

**Authors:** Ruoyu Luo, Lin Ye, Chenyang Tao, Kankan Wang

**Affiliations:** 1 School of Life Sciences/Center for Computational Systems Biology, Fudan University, Shanghai, China; 2 State Key Laboratory of Medical Genomics, Rui-Jin Hospital Affiliated to Shanghai Jiao Tong University School of Medicine, Shanghai, China; 3 Shanghai Center for Bioinformation Technology, Shanghai, China; Universite de Sherbrooke, Canada

## Abstract

Due to the complexity of biological systems, simulation of biological networks is necessary but sometimes complicated. The classic stochastic simulation algorithm (SSA) by Gillespie and its modified versions are widely used to simulate the stochastic dynamics of biochemical reaction systems. However, it has remained a challenge to implement accurate and efficient simulation algorithms for general reaction schemes in growing cells. Here, we present a modeling and simulation tool, called ‘*GeneCircuits*’, which is specifically developed to simulate gene-regulation in exponentially growing bacterial cells (such as *E. coli*) with overlapping cell cycles. Our tool integrates three specific features of these cells that are not generally included in SSA tools: 1) the time delay between the regulation and synthesis of proteins that is due to transcription and translation processes; 2) cell cycle-dependent periodic changes of gene dosage; and 3) variations in the propensities of chemical reactions that have time-dependent reaction rates as a consequence of volume expansion and cell division. We give three biologically relevant examples to illustrate the use of our simulation tool in quantitative studies of systems biology and synthetic biology.

## Introduction

One of the main objectives in systems biology is to quantitatively understand the behavior of biological systems, particularly from a dynamic aspect. Based on the dynamic information of biological systems, synthetic biology allows for the rationale design of artificial gene circuits. Improvements in various “-omics” and biophysical technologies have provided an accumulation of data; this wealth of knowledge and experimental data enables simulations of gene regulatory networks in an increasingly accurate manner [1], [2], [3], [4], [5], [6], [7], [8], [9], [10]. More importantly, the quantitative models could help us in understanding the general principles regarding how gene regulation systems are operated [10], [11], [12], [13], [14], [15]). The classic stochastic simulation algorithm (SSA) by Gillespie and its later developments are widely used to simulate the stochastic dynamics of well-stirred biochemical systems [16], [17], [18]. The algorithm is easy to implement on well-stirred reaction volumes involving zeroth-, first- and second-order elementary reactions that have contributed to its popularity [16], [19]. However, even the simplest gene regulatory circuits in bacteria possess common features that are not trivial to implement in a general simulation tool.

The first feature is the cell cycle-dependent gene expression level. It is caused by variations in gene dosages (due to the gene’s position in the chromosome) and sometimes has important physiological significance [20], [21]. When a chromosomal gene position is near the replication origin site, the replication fork passes this position earlier and doubles the gene copy number, thereby causing this gene to have a higher average dosage in one cell cycle. Conversely, if a gene is far away from the replication origin site, the replication fork passes its position later, resulting in a lower average dosage in one cell cycle [22]. For the *E. coli* gene expression system, a higher gene dosage means the opportunity to express more proteins [20]. Also several bacterial species have overlapping cell-cycles such that chromosome replication may be initiated in the grandmother cell, to be able to finish a round of replication and chromosome segregation before cell division. This implies that a single gene may have up to eight copies in one cell at high growth rates [23], which may significantly buffer the low copy number fluctuations in gene expression.

The second is the variations in the propensities of chemical reactions as a consequence of volume expansion and cell division. The volume of *E. coli* increases exponentially and divides into two daughter cells about 20 minutes (the D-period) after the replication of the chromosomes is completed. The effects of the gradual change in cell volume influence the propensity of bi-molecular reactions. For example, the chance for two molecules to find each other before cell division is doubled than the chance after cell division, assuming that these two molecules would end up exclusively in the same cell.

The last but not least feature is the time delay between the regulation and synthesis of proteins. It is not always possible to break down all reactions into elementary steps. Since synthesis of macromolecular polymers such as RNA and proteins involves a large number of sequential synthesis steps and the synthesis time is not exponentially distributed, it is often misleading to approximate all these steps with a single elementary reaction. Thus, it is highly desired for the multi-step synthesis processes to introduce a time delay between the start of synthesis and the emergence of a functional macromolecule. Several reports have shown that time delays account for memory ability and instability in biological systems [24], [25], [26], [27], [28], especially in those cases with highly stochastic behavior in gene regulation systems when molecules exist in low copy numbers [29].

Simulation methods that consider either volume expansion or time delays in analyzing reaction systems have been described previously [30], [31], [32]. To our knowledge, there is still a lack of methods that can consider aforementioned features simultaneously. Moreover, there is a great need to take into account the peculiarities of dynamic gene dosage effects in cells with overlapping cell cycles. In this study, we integrate these features together and package them in a user-friendly simulation tool ‘*GeneCircuits*’.

## Methods

### Consideration of Periodic Gene Dosage Changes and Cell Division

The program takes a deterministic generation time of the cells and the positions of the relevant genes as inputs and calculates dynamic changes in gene-dosage throughout the cell cycles. To make the implementation feasible, we made the following assumptions: the chromosome replication time (the C-period) is constant (*i.e.*, 40 minutes [33]); the replication fork moves at a constant speed from the origin of replication to the terminus; the bacteria are divided after 20 minutes following the completion of chromosome replication (the D-period) [33], [34], [35]. After each simulated event, the algorithm updates the position of each replication fork and calculates the current gene dosage of each gene. Once a fork moves through the gene position on the chromosome, the copy number of this gene is doubled, and the new instance of the gene copy is created and maintained by the algorithm. Moreover, the algorithm also creates corresponding reactions for the new gene copy, including its own transcription process and corresponding transcription factor association and dissociation from the promoter for this gene. These new reactions are then pushed into the reaction queue, giving them the opportunity to be chosen later. It is noteworthy that the regulation of new copies of this gene is independent.

Cell division is an important source of noise in the *E. coli* system [36], [37]. Upon division, the algorithm distributes all free molecules between the mother cell and the daughter cell according to a binomial distribution function [38]. Furthermore, the algorithm keeps track of the initial conditions and relationships between bacteria after cell division, i.e. if one daughter cell gets more, the other gets less. We used this information to rebuild the lineage tree. Based on these data, the software provides analysis methods to mine dynamic information on the development of *E. coli* micro-colonies and to analyze correlations with concentrations over several generations. During cell division, for example, the states of the individual promoters are inherited by the daughter cells, whereas freely diffusing molecules and complexes are randomly partitioned. This function makes it possible to study how epigenetic states are inherited throughout the cell linage tree.

### Cell Volume Increase and Time Delays in the Simulation of the Gene Expression Process

Since cell volume growth has a great influence to the time-dependent reaction in the system, and since multi-step synthesis processes introduces time delays in the dynamics of gene expression processes, we thus integrated time delays and cell volume increase into the framework.

A typical reaction system includes both time-dependent and time-independent reactions. Therefore, we adopted the algorithm by Lu et al. [30] to sample the next reaction event in the system. Consistent with the previous notations, we denote the total rate of reactions with time-dependent propensities by 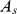 and that without time-independent propensities by 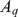. In contrast to the classic Gillespie algorithm approach that calculates the propensity of a combination reaction, we obtain the current concentration of each element of this combination reaction at each time step. Equation (1) is used to calculate the probability of all association reactions and equation (2) is used to calculate the propensities of the remaining channels.
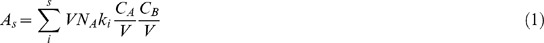


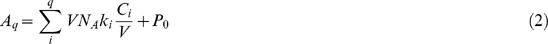
>

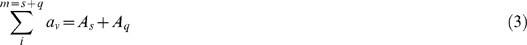



In equations (1), (2) and (3), 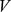 is the current volume of the bacterium, 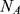 is the Avogardro’s number, k is the reaction constant in the sense of classical kinetics, 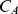 is the copy number of one reactant, 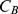 is the copy number of the other reactant, 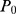 is the total rate of zeroth-order reactions, 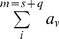
is the total propensity of this system, and 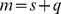 is the total channel number.

To estimate the time step, we introduce the following definition
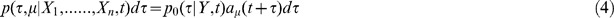
where 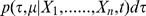 is the probability that, given the state 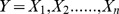 at time 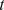, the reaction 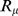 will occur in the infinitesimal time interval 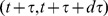, and 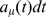 is the probability that reaction 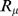 will occur within the interval 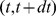 under current state: 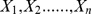.

When no reaction occurs during 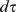, we can calculate the probability in the situation as following:
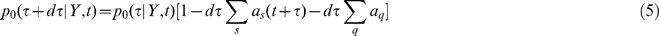



Using the initiation condition: 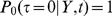, the 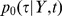 can be derived to:
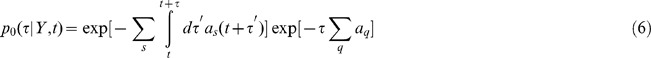



Through combining equations (4) and (6), 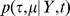 can be described as following,
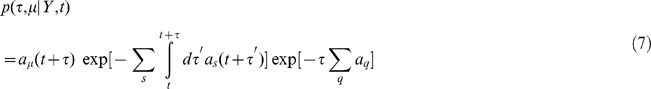



Since the cell volume grows following the exponential law 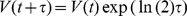, we can obtain the probability of any reaction occurring between time 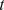 and 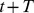 using the equation (8).
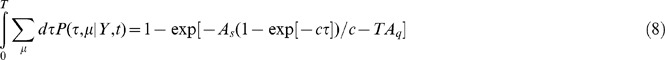



By generating a uniform random number and letting it be equal to the right side of equation (8), we use the equations in (9) to sample when the next reaction will occur.
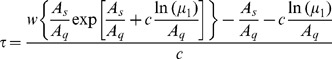



if 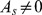 and 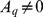


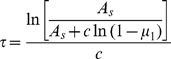



if 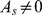 and 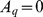


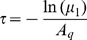


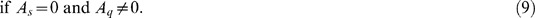



In equation (9), 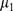 is a uniform random number, 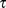 is the time step, 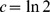, and 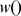 is the Lambert function, which is the solution of the equation of 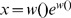.

Using equations (1)–(9), the algorithm samples the interval in time to the next reaction. Meanwhile, the algorithm also checks whether there is a reaction with time delay in the delayed queue or a gene replication event scheduled during the interval. If there is a scheduled delayed reaction or a replication event that will occur during the interval at time 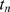, the algorithm updates the system time to 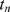. After this, a new event time is sampled using equation (9). However, if the selected reaction is a time-delayed reaction scheduled to occur at 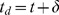, where 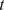 is the current system time and 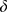 denotes the delayed time of this reaction, the algorithm pushes the reaction into a queue that has been created for the temporary storage of time-delayed reactions, and the reaction product will not be released until the system time reaches 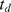.

If the biochemical reactions system does not have time delays and the interval between adjacent reactions is large enough, the Lambert function is appropriate to be used to estimate the interval time of two adjacent reactions. However, in the systems with time-dependent and delayed time reactions, the precision of calculation of Lambert function is critical to the correctness of algorithm. For example, there is a delay reaction in the reaction queue and has been scheduled within the time interval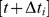. In most of cases, 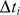 is a small interval. If the accuracy of calculation of Lambert function is dissatisfactory, the algorithm will return a less accurate result, which leads 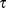 always bigger than 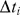 and eventually induces the algorithm to only choose delay reactions in the reaction queue as the next reaction until the queue is empty. However, there are no chances for other channels to be chosen.

### The Flow Chart of the Algorithm

The flow chart of the algorithm is listed below in detail.

1. Calculate the expected time point of the replication initiation and termination of the chromosome. For each gene, estimate the expected replication time point during the whole simulation time. Push all of these time points into a queue, which stores all of the scheduled delayed reactions and events, and then sort all of the elements in this queue.
2. Input value constants for each reaction: 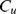, 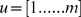, initial state 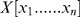, set 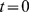 and the reaction counter 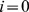, where 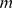
is the number of reactions and 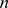
is the number of reactants.
3. Calculate 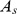, 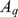 and their sum using equations (1), (2) and (3).
4. Generate uniformly distributed random numbers: 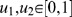;
5. Use equation (9) to estimate 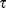, which denotes the time interval from the current time until the next reaction.
6. Check the queue to see if there are delayed reactions or if an event of gene replication is scheduled during the time interval 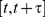. If YES and the top element of the queue is a scheduled delayed reaction, the algorithm updates the system state to the chosen reaction channel and the system time to 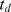, pops this chosen reaction from the queue, increases the system volume, set 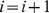 and go to step 3. Otherwise, if YES and the chosen reaction is a gene replication event, the algorithm updates the state of the gene promoter, sets the system time to the replication time of this gene, doubles the gene copy number, creates the corresponding reactions, fills them into the reaction list, pops the chosen event from the queue, increases the system volume, sets 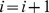 and switches to step 3. If NO, go to step 7.
7. Take 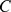 to be the integer for which: 
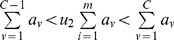
, where 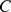 is the index of the next chosen reaction. If the selected reaction is delayed, push it into the queue, sort the queue, increases the system volume, and go to step 3.
8. Update the system state according to the channel for the next reaction, advance the system time to 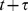, increase the system volume and let 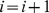. Go to step 3.

### Implementation of the Software

**Figure 1:**
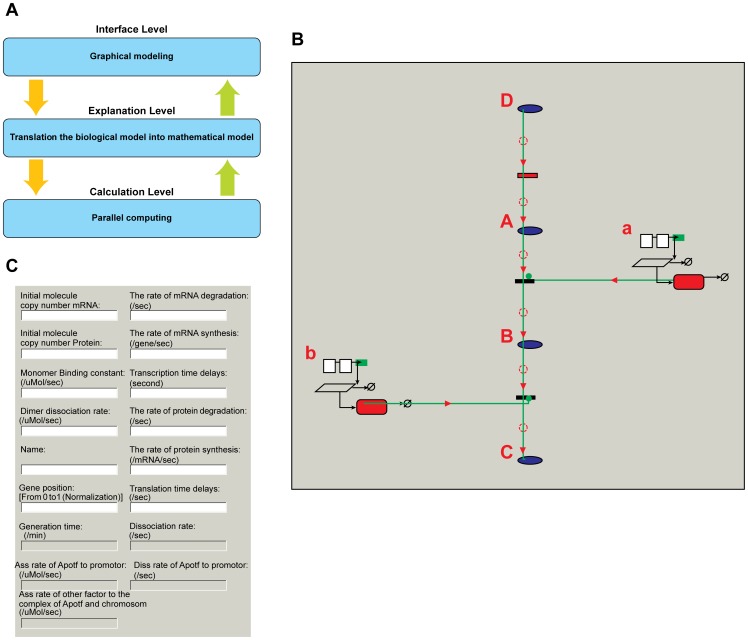
Schematic diagram of the software architecture. (A) The tool contains three logical levels: the interface level, explanation level and calculation level. The interface level is an editor to build biological models and integrate necessary information and parameters. The explanation level is a complier to understand the user’s biological model and translate it into the mathematical model. The calculation level is a computational element to calculate the model and return the results. (B) The interface of building the model and setting parameters. With a user-friendly interface, the tool provides an instant visual bio-model building environment. For user convenience, *GeneCircuits* chose standard icons to present biological elements. For example, a gene icon is represented by a set of standard sub-symbols, including two regulatory domains (white square), one mRNA (hollow parallelogram) and one corresponding protein (solid red rounded quadrilateral). Users can define the various roles of each element in the system. Based on the Petri net representation, each reaction has a horizontal line, which is a representation of the reaction. By double-clicking this horizontal line, users can set up parameters of the reaction. (C) The user interface for setting parameters.

We developed a user-friendly C++ software called *GeneCircuits*. This tool is designed with three logical levels (see **Figure 1**): the interface level, the explanation level and the calculation level. The interface level is a graphical biological model editor used to reconstruct gene regulation systems. At this level, the relevant information is integrated to build a biological model. The explanation level is a model compiler with the built-in functions to understand the network and translate it into the corresponding modified Gillespie model. The calculation level calculates the model parameters and, if required, can also distribute the task on several cores. This modular design not only makes it easier to maintain, but also makes it more flexible to incorporate multilevel parallel computation.

From the viewpoint of software engineering, *GeneCircuits* is a distributed memory parallel system, which takes advantage of multithreading technology from the Boost thread library (http://www.boost.org/). Each cell is simulated by one thread. Each time a daughter cell is created, a matched thread is accordingly created. Additionally, the tool remembers the hierarchy relationship between them. With the multithread technology, *GeneCircuits* not only efficiently implements the underlying algorithm, but also holds great promise in further simulating the communication and cooperative behavior between bacteria. In the case that the simulation of complex networks requires huge calculation tasks, the users can also utilize the grid function of the tool. This grid function distributes the tasks to the client notes so as to further improve the efficiency.

The software package, source codes, manual and teaching videos, along with some example models, can be found at (http://ccsb.fudan.edu.cn/genecircuits/). The teaching videos including all of the basic operations and some of the advanced operations can help users learn the software package. Also, comprehensive software test cases are provided, which have been used to detect bugs so as to prove the rationality of the simulation results. Users can download the test cases and the corresponding stochastic and deterministic models from the above website as well. Since our software takes into account numerous specific biological details and integrates biologically oriented designs, the efficiency of our tool may not be as high as tools tailored to specific situations.

### Biologically Oriented Features of *GeneCircuits*

Currently, there are several popular simulation tools available, such as E_Cell [39], Dizzy [40], Virtual Cell [41], CellLine [15], STOCHSIM [42] and Dynetica [43]. These tools are user-friendly and can work well on many biochemical networks and simplified gene regulatory processes. However, these tools simplify gene regulatory processes and are not so easily modified for investigation of gene regulation or biochemical networks with cell cycle dependent noises, time delay and gene dosage effects. Instead, our tool presented here is suited to stimulate cell cycle dependent gene regulation, because we have integrated the following biologically oriented designs into the framework:

Firstly, our tool considers the relationship among transcription factor (TF), its cofactors and target genes. The TF affects the expression of its downstream target gene via association or dissociation to specific binding sites located in the promoter of the target gene. Cofactors influence the affinity of the TF to its target gene through binding or unbinding TF’s active site, which will eventually regulate the expression of downstream target genes of TF. *GeneCircuits* provides the functionality and interfaces that allow users to keep track of the activity states of promoters in the presence of transcription factors that themselves can be regulated transcriptionally or metabolically by binding small molecules.

Secondly, our tool provides the functionality to define the cooperative law and thus permits the simulations of the dynamic binding affinity adjustment. This design in *GeneCircuits* is important since most of genes are cooperatively regulated by one or more TFs.

Thirdly, in some case the extrinsic noise influences the gene expression in bacteria [37], [44], [45]. To approximate the effect of extrinsic noise, our tool can multiply Ornstein-Uhlenbeck noise to the reaction rate constants. The extrinsic noise is assumed to be the same for all genes and have cell cycle-dependent autocorrelation time and noise intensity [46], [47]. Also, it can provide the interface by which users can choose to apply or not to impose simultaneously the activity environmental fluctuations on many parameters of the network.

## Results

To demonstrate the capability of *GeneCircuits* for exploring complicated phenomenon of gene regulation systems, we applied this tool to three biological model systems. Using a negative auto-regulation system with or without time delay respectively, we first showed the capability of the tool for demonstrating identified cell dynamics. Then, we illustrated epigenetic inheritance based on a bitable switch in a micro colony. Finally, we demonstrated the effect of gene dosage dependent gene expression with a metabolic flux balance model. The three examples represent major features of our proposed algorithm. The model files for simulating these examples are also available as a part of the software (i.e., in the ‘/model’ folder after *GeneCircuits* is installed). Below we described these examples together with the biological significance.

### Simulation of Time Delay Processes in a Negative Auto Regulation System

First, we investigated the performance of *GeneCircuits* in a negative auto-regulation system. The model contains four elements: the gene promoter, mRNA, protein and dimer. The dimer is created by proteins and has the capability to bind to its own promoter. Once the dimer binds to the gene promoter, the transcription rate of the gene is decreased by a factor of 10. The bound TF dissociates from the promoter after the replication fork passes the gene on the chromosome. To let the promoter bind again quickly, we set high values for the dimer binding rate. The parameters of the system are listed in Table 1.

**Table 1:** The rates of parameters used in the auto-regulation systems.

	Symbol	Value	Unit
Transcription rate	*a*	1	/*gene*/sec
Translation rate	*b*	1	/*mRNA*/sec
mRNA degradation rate	*r_1_*	2	1/sec
Protein decay rate	*r_2_*	0.01	1/sec
Dimer production rate	*c*	1	*µM*/sec
Dimer dissociation rate	*d*	1	1/sec
Rate of dimer binding to the promoter	*K_1_*	100	*µM*/sec
Rate of dimer dissociation from the promoter	*K_2_*	0.1	1/sec

We built the corresponding deterministic mathematical model (see Matlab code 1, in the Text S1) for this system as follows:
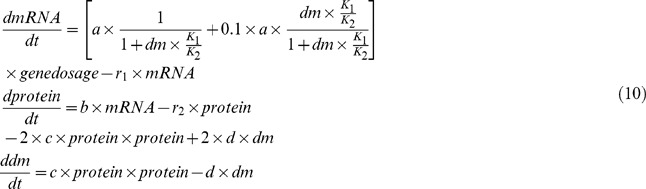



In equation (10), we set the gene dosage 0.003 µM (about two gene copies/cell). The variables *mRNA*, *Genedosage*, *protein* and *dm* refer to the concentration of the mRNA, gene copy, protein and dimer in the system, respectively. The initial values of these variables are set to zero.

**Figure 2:**
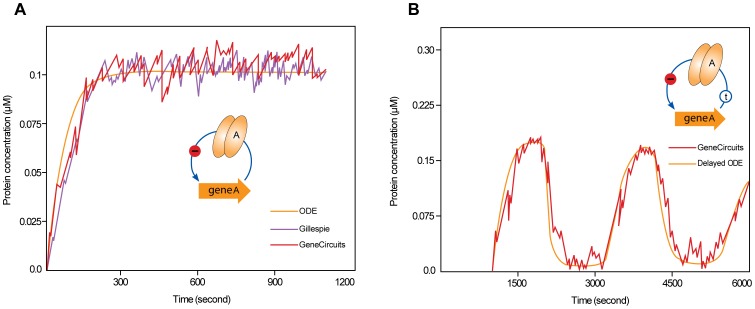
Simulation of a negative auto-regulation system with or without time delay. (A) When the simulation of the system without time delay is performed, the curves from the deterministic equations and the classic Gillespie method are smooth and approximately equal. The protein (monomer) copy number of a time series is generated from the deterministic equations (ODE, yellow curve), the classic Gillespie method (Gillespie, blue curve) and our stochastic algorithm (*GeneCircuits*, red curve). The curve of *GeneCircuits* is slightly higher than those of the two other simulations. (B) Oscillations in the protein concentration are induced at a longer time delay (1000 seconds).

To test the implementation of the algorithm, we first applied *GeneCircuits* to the model without time delay and compared the results from our algorithm with those from the classic SSA (see Matlab code 2 in the Text S1) and the ordinary differential equation (ODE) methods. We simulated the classic Gillespie model with two copies of a gene. In the model of our algorithm, we set a long generation time (200 min) that caused the cell volume to slowly increase so that cell growth could almost be neglected. We set the gene position near the replication origin site on the chromosome, leading to a gene dosage approximately equal to the two above models. As shown in **Figure 2A**, the results from the classic Gillespie model are identical to those from the ODE result. The results from our modified algorithm are similar to, but slightly higher than, those from the ODE and classic Gillespie methods. The higher results from our algorithm are as expected; we accounted for the cell growth volume in our algorithm, and thus the probability of dimer binding to the target promoter was modulated gradually by time.

Next, we compared the results from the models with time delay, that is, the delayed ODE model and our modifier model. Using the same parameters as those in the above models, we introduced a long time delay (1000 seconds) into the translation process and simulated the model for 400 min (two cell cycles). Notably, it is experimental but without indicated biological significance. As shown in **Figure 2B**, we observed obvious oscillations for both models considering time delay. The long delayed time in the translation process brings the system far away from its stable point, which results in oscillation around the stable point to create a limit cycle oscillator [48].

### The Impact of Heterogeneity in Micro-colonies

**Figure 3:**
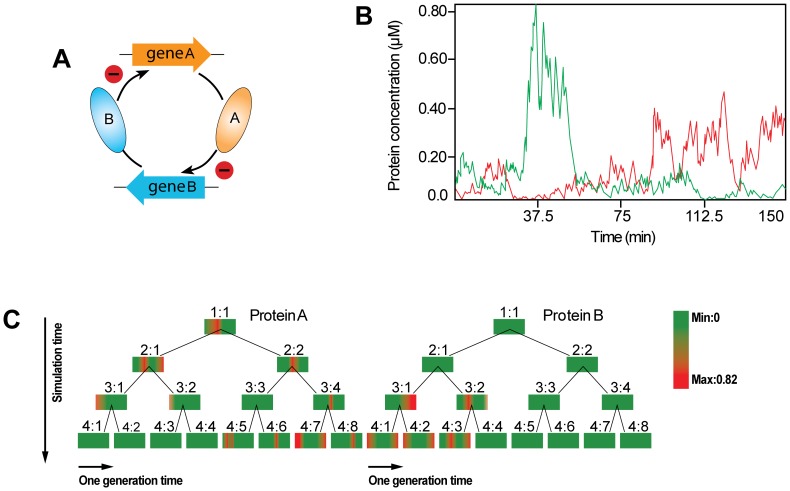
Bi-stable system in a double-negative feedback loop. (A) A schematic illustration of a double-negative feedback loop is shown. Protein A acts as a repressor of gene B, and protein B represses the expression of gene A. (B) For the parameters given in the model, the system is bi-stable. (C) Two lineage trees (left and right) describe the distribution dynamics for the expression of Genes A and B. The color bar represents the concentration of the protein, and each bar represents the profile of one protein of one bacterium during one cell cycle. For the unsynchronized initial states, the lineage trees of the expression of Gene A and B display inverse correlations.

Under natural conditions, the state of each bacterium is sometimes unsynchronized [22], [49]. To approximate the natural conditions, we further considered a model: two genes can repress each other and their corresponding product proteins have equal binding abilities to their target genes (**Figure 3A**). As shown in **Figures 3B** **,** at the beginning of the simulation, the state of gene expression is random. Because the regulator circuit is bistable, the pre-existing heterogeneity is then passed down to later generations. To lay out the sustainable bi-stability, we displayed two lineage trees describing the protein concentrations during the entire simulation time in **Figure 3C**. By comparing two lineage trees, we found clear asynchrony or inverse correlations between the expression levels of the two genes.

### Large Fluctuations of the Metabolite Pool Caused by Dynamical Gene Dosage Effects

With the rapid advancement of synthetic biology, many studies have designed artificial gene circuits or metabolic pathways to explore the general law of biological networks, thereby improving the production rate of biochemical products. However, as far as we know, few studies have considered the location of a gene on the chromosome. To do so, we applied our modified algorithm to a dynamic metabolic flux balance model, and demonstrated that our modified algorithm is able to simulate the gene expression change affected by the chromosomal location of a gene, and can also simulate the consequence of this change to artificial system.

**Figure 4:**
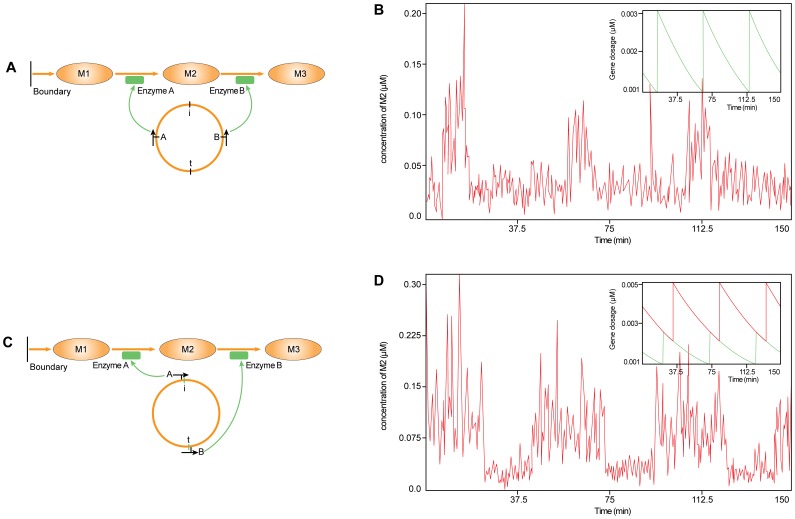
The fluctuation of the metabolite concentration due to the gene position of the enzyme on the chromosome. (A) The biological model with the balanced flux of metabolite M2. There are two enzymes, A and B, and three metabolites, M1, M2 and M3. M1 can be taken up from the surroundings. Enzyme A and enzyme B can catalyze the input flux and output flux of metabolite M2, respectively. In this figure, we use “i” and “t” to represent the starting point of chromosome replication and termination, respectively. (B) If two enzymes have the same gene position on the chromosome, the average rate of the input flux and output flux are equal. The fluctuation of M2 concentration is only due to the replication of the genes and bacterial volume growth. The insert illustrates the gene dosage. (C) The model of unbalanced flux of M2. There is a long distance between the gene positions of two enzymes on the chromosome. (D) The difference in the temporal expression of two enzymes caused the inequality of the average input and output flux and enhanced the fluctuation of M2 concentration. The insert shows the gene dosage of A (green curve) and B (red curve).

We set up a simple metabolic network (**Figure 4A** **)**, in which the relative concentration of enzymes has a great impact on the metabolism levels since the enzymes operates close to saturation [50]. In this metabolic network, the system can uptake metabolite M1 from the surrounding environment, enzyme *a* catalyzes metabolite M1 to metabolite M2, and enzyme *b* catalyzes metabolite M2 to metabolite M3. The kinetic parameters for the two enzymes were set to be equal and the full parameters used in this metabolic system were listed in Table 2.

**Table 2:** The parameters used in the metabolic system.

	Symbol	Value	Unit
Update rate of M1	*inputflux_M1_*	100	*µM*/sec
Output of M1	*outputflux_M1_*		*µM*/sec
Utilize rate of M1 and M3	*r_3_*	1	1/sec
mRNA degradation rate	*r_1_*	1	1/sec
Enzyme decay rate	*r_2_*	0.01	1/sec
Enzyme Kcat value	*Kc*	10	1/sec
Enzyme Km value	*K_m_*	1	*µM*
mRNA synthetic rate	*a*	1	/*gene*/sec
Enzyme synthetic rate	*b*	1	/*mRNA*/sec
Dilution rate	*μ*	0.0139	NA

Using the Flux balance equations [51], we described the dynamics of the system as equation (11).

In equation (11), the variables: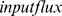, 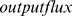 and 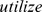 describe the input flux, output flux and utilized rate of each metabolite, respectively. We applied the Michaelis-Menten equation to define the input and output fluxes [52], [53]. The initial values of the three metabolites are set to 50 molecules, and the initial values of protein and mRNA are set to 100 and 10 copies, respectively. We set the system generation time to 50 minutes and studied the system in two steps.

First, two genes were positioned symmetrically at the middle of the chromosome. When the expression level of one gene changes periodically along with the doubling and halving of the gene copy number, the temporal dynamics of both genes display the same characteristics; thus, the input flux of metabolite M2 and the output flux of metabolite M2 maintain a general balance. Under this balanced condition, the copy number of metabolite M2 stays at a certain level, and fluctuations in the concentration of metabolite M2 will be caused by cell volume increase and gene replication. The results are shown in **Figure 4B**.

Second, gene a was moved in the direction of the replication origin, while gene b was moved in the opposite direction toward the telomere (**Figure 4C**). Thus, at the beginning of each generation, gene a has two copies, and its gene copy number will double 40 minutes later. However, gene b has only one copy at the beginning of each generation and will double 30 minutes later. The difference between the expression levels of the enzymes leads to an unbalanced input and output flux of metabolite M2. Although the system volume will grow and the cell will divide, the discrepancy of fluxes gives rise to the accumulation of metabolite M2, eventually causing the concentration of metabolite M2 to stay at a higher level much longer. The results are shown in **Figure 4D**. In addition to the *Genecircuits* model, we also applied the SSA method (please see Matlab code 3 in the Text S1) to this dynamic Flux Balance system and compared the differences between the results of the two models.
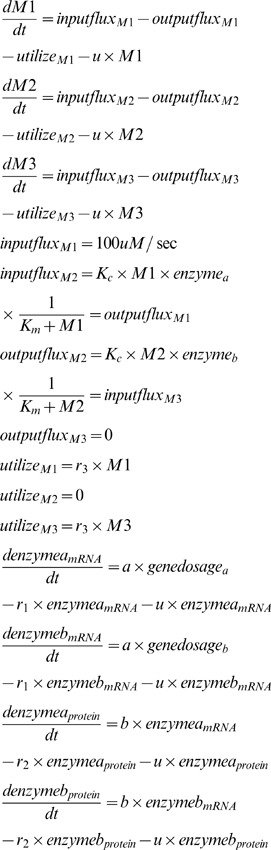
(11)


Even though our modifier model gives better results, it should be emphasized that this is still a simplified and idealized model. In a real metabolic system, excessive accumulation and consumption are a waste of valuable resources and are even deleterious to the function of a system in some cases, particularly under perturbation conditions [49], [54]. Biological systems develop delicate mechanisms, such as negative feedback of gene regulation systems [55] and cooperation between metabolic pathways, to avoid excessive accumulation and consumption [56] and to allow for supporting systems to function optimally. Usually, *E. coli* is genetically engineered by inserting some external enzymes gene on its chromosome. For this modified strain, our method is able to mimic such real scenario by simulating the effect of chromosomal location, which can be used to improve capability of the metabolic pathway by modification of the gene locations.

## Discussion

In this study, we extended the classic SSA algorithm to the one that simultaneously takes into account key features during gene regulation of bacterial cells with overlapping cell cycles. Also, we developed the simulation tool *GeneCircuits*, freely available to the public. The modified algorithm and the biologically oriented software provide a new way to investigate the dynamics of *E. coli* gene regulation and biochemical systems.

With the rapid improvements in synthetic biology [57], there is a great need to test *in silico* designs under “realistic” as much as possible [58]. Enzyme genes, TF genes and other elements have different time delays in translation and transcription processes, each with different associated properties. They are positioned at different places on the chromosome. As chromosome is replicated and cell volume increases, the gene expression levels and the propensity of the time-dependent reactions differ at the different cell cycle phases. Moreover, the time delay in biological processes further complicates the dynamics of the system. These complex and coupled factors should be considered simultaneously in the ‘ideal’ design to make sure the functions appropriately. With our algorithm and the tool *GeneCircuits*, users can meet their desired need for the biological system of interest: 1) design biological elements with a certain expression ability, 2) consider time delay in translation and transcription processes, and 3) modulate the chromosomal gene position to test the response of the system. Therefore, users can choose the optimized gene arrangement on the chromosome with the best tradeoff between system function and gene propensity.

As compared to an experimental tube, our simulation method accounts for several key aspects of gene regulation that needs to be modeled differently in a living bacterial cell. Even though, there are still a great number of possible biophysical factors left to be considered for accurate *ab initio* simulation of intracellular processes. To identify these factors, we need to add complexity over test tube chemistry one level at the time until we reach convergence with experimental measurements in living cells. The central features for exponentially growing microorganisms growing with overlapping cell cycles that we have simulated in this study represents an important step towards this path.

## Supporting Information

Text S1 Supplementary matlab codes.
(DOC)
