# Supplementary material for: Simulation of E. coli Gene Regulation including Overlapping Cell Cycles, Growth, Division, Time Delays and Noise
Source: PLoS One. 2013 Apr 26;8(4):e62380. doi: 10.1371/journal.pone.0062380 (PMC3637171; doi:10.1371/journal.pone.0062380)
Supplement: Text S1 — Supplementary matlab codes. (DOC) [file pone.0062380.s001.doc]

%%Matlab code 1：

a =1;

b =1;

c =1;

d =1;

r1 = 2;

r2 = 0.01;

k1 = 100;

k2 = 0.1;

options = odeset('RelTol',1e-4,'AbsTol',[1e-4]);

[T,Y] = ode45(@rigid,[0 1000],[0,0,0],options,a,b,c,d,r1,r2,k1,k2);

figure(1);

plot(T,Y(:,1)*602,'-');hold on;

figure(2);

plot(T,Y(:,2)*602,'-');hold on;

figure(3);

plot(T,Y(:,3)*602,'-');hold on;

sol = dde23(@rigiddelay,[1000],[0 0 0],[0 6000],options,a,b,c,d,r1,r2,k1,k2);

figure(4);

plot(sol.x,sol.y(1,:)*602,'-');hold on;

figure(5);

plot(sol.x,sol.y(2,:)*602,'-');hold on;

figure(6);

plot(sol.x,sol.y(3,:)*602,'-');hold on;

function dy = rigid(t,y,a,b,c,d,r1,r2,k1,k2)

dy =zeros(3,1);

dy(1) = (a*(1/(1+y(3)*(k1/k2))) + 0.1*a*(y(3)*(k1/k2)/(1+y(3)*(k1/k2))))*0.003 - r1*y(1);%mRNA

dy(2) = b*y(1) -r2*y(2)-2*c*y(2)*y(2)+2*d*y(3);%protein

dy(3) = c*y(2)*y(2) - d*y(3);%dimer

function dy = rigiddelay(t,y,z,a,b,c,d,r1,r2,k1,k2)

ylag1 = z(:,1);

dy = zeros(3,1);

dy(1) = (a*(1/(1+y(3)*(k1/k2))) + 0.1*a*(y(3)*(k1/k2)/(1+y(3)*(k1/k2))))*0.003 - r1*y(1);%mRNA

dy(2) = b*ylag1(1) - r2*y(2)-2*c*y(2)*y(2)+2*d*y(3); %protein

dy(3) = c*y(2)*y(2) - d*y(3);

%Matlab code 2：

%%Please change the file name to "do.m"

%%Please change the file name to "do.m"

%%Please change the file name to "do.m"

%%Please change the file name to "do.m"

%%Please change the file name to "do.m"

%%Please change the file name to "do.m" Ruoyu Luo

R0 = 2;

R = 0;

Arr = 100;

mRna = 10;

dimer = 1;

transcription_rate0 = 1;

transcription_rate1 = 0.1;

mRna_decay_rate = 2;

translation_rate = 1;

Protein_decay = 0.01;

V = 1e-15;

A= 6.02e+17;

tstop = 1000;

t=0;

c=1;

%%

binding_rate = 100;

dissociation_rate = 0.1;

Protein_dimer = 1;

Protein_dimerdiss = 1;

t=0;

c=1;

tstop = 4000;

R0 = 2;

R = 0;

Arr = 100;

mRna = 10;

T0 = [1:10000]; %zeros(1);

y00 = [1:10000];%zeros(1);

y01 = [1:10000];%zeros(1);

y02 = [1:10000];%zeros(1);

y03 = [1:10000];%zeros(1);

y04 = [1:10000];%zeros(1);

rand('state',sum(100*clock));

index = 0;

while t <= tstop

index = index+1;

b =[0 0 0 0 0 0 0 0 0];

b(1) = (V*A)*binding_rate*(dimer/(V*A))*(R0/(V*A)); %bind

b(2) = dissociation_rate*(R); %diss

b(3) = transcription_rate0*(R0); %transcription 0

b(4) = transcription_rate1*(R); %transcription 1

b(5) = mRna_decay_rate*(mRna); %mRna decay

b(6) = translation_rate*(mRna); %protein produce

b(7) = Protein_decay*(Arr); %protein decay

b(8) = (V*A)*Protein_dimer*(Arr/(V*A))*(Arr/(V*A)); %dimer create

b(9) = Protein_dimerdiss*(dimer); %dimer diss

astr = b(9)+b(8)+b(7)+b(6)+b(5)+b(4)+b(3)+b(2)+b(1);

if~astr, break,end

tau = -1/astr*log(rand);

rt = astr*rand;

totalb = 0;

for k = 1:9

totalb = totalb+b(k);

if(totalb >= rt)

break;

end

end

u = k;

if(u == 1)

R0 = R0 - 1;

R = R + 1;

Arr = Arr -1;

dimer = dimer -1;

end

if(u == 2)

R0 = R0 + 1;

R = R - 1;

dimer = dimer + 1;

end

if(u == 3)

mRna = mRna + 1;

end

if(u == 4)

mRna = mRna + 1;

end

if(u == 5)

mRna = mRna - 1;

end

if(u == 6)

Arr = Arr + 1;

end

if(u == 7)

Arr = Arr - 1;

end

if(u == 8)

if(Arr >= 2)

dimer = dimer + 1;

Arr = Arr - 2;

end

end

if(u == 9)

if(dimer >= 1)

Arr = Arr + 2;

dimer = dimer - 1;

end

end

t=t+tau;

T0(index) = t;

y00(index) = R;

y01(index) = R0;

y02(index) = Arr;

y03(index) = mRna;

y04(index) = dimer;

end

figure(6);

plot(T0(1:index),y00(1:index),'-');title('\bf\fontsize{14}A. R'); hold on;

figure(7);

plot(T0(1:index),y01(1:index),'-'); title('\bf\fontsize{14}B. R0');hold on;

figure(8);

plot(T0(1:index),y02(1:index),'-'); title('\bf\fontsize{14}C. Arr');hold on;

figure(9);

plot(T0(1:index),y03(1:index),'-'); title('\bf\fontsize{14}D. mRna');hold on;

figure(10);

plot(T0(1:index),y04(1:index),'-'); title('\bf\fontsize{14}D. dimer');hold on;

%%Matlab code 3

transcription_rate = 1;

mRna_decay_rate = 0.1;

translation_rate = 1;

Protein_decay = 0.1;

K1 = 100;

Adecay = 1;

K2 = 0.00000000000001;

K3 = 0.00000000000001;

Cdecay = 100;

Kcat = 10;

Km = 0.1;

InputfluxofA = 100;

t=0;

c=1;

tstop = 2400;

Arr0 = 100;

mRna0 = 10;

Arr1 = 100;

mRna1 = 10;

A = 50;

B = 50;

C = 50;

T0 = [1:10000]; %zeros(1);

y00 = [1:10000];%zeros(1);

y01 = [1:10000];%zeros(1);

y02 = [1:10000];%zeros(1);

y03 = [1:10000];%zeros(1);

y04 = [1:10000];%zeros(1);

y05 = [1:10000];%zeros(1);

y06 = [1:10000];%zeros(1);

rand('state',sum(100*clock));

index = 0;

while t <= tstop

index = index+1;

b =[0 0 0 0 0 0 0 0 0 0 0 0 0 0 0];

if(t < 1200) % 20 mins

R0 = 2;

R1 = 1;

b(1) = K2; %B input flux, don't catalyzed by enzyme a

b(2) = K3; %C input flux, don't catalyzed by enzyme b

b(3) = transcription_rate*(R0); %transcription a

b(4) = transcription_rate*(R1); %transcription b

b(5) = mRna_decay_rate*(mRna0); %mRna decay a

b(6) = mRna_decay_rate*(mRna1); %mRna decay b

b(7) = translation_rate*(mRna0); %enzyme a produce

b(8) = translation_rate*(mRna1); %enzyme b produce

b(9) = Protein_decay*(Arr0); %enzyme a decay

b(10) = Protein_decay*(Arr1); %enzyme b decay

b(11) = ((V*A)*Kcat*(Arr0/(V*A))*(A/(V*A)))/(Km+(A/(V*A))); %B input flux catalyzed by enzyme a

b(12) = ((V*A)*Kcat*(Arr1/(V*A))*(B/(V*A)))/(Km+(B/(V*A))); %c input flux catalyzed by enzyme b

b(13) = Adecay*A;

b(14) = Cdecay*C;

b(15) = InputfluxofA;

end

if(t > 1200 && t < 2400) % dosage double

R0 = 4;

R1 = 2;

b(1) = K2; %B input flux, don't catalyzed by enzyme a

b(2) = K3; %C input flux, don't catalyzed by enzyme b

b(3) = transcription_rate*(R0); %transcription a

b(4) = transcription_rate*(R1); %transcription b

b(5) = mRna_decay_rate*(mRna0); %mRna decay a

b(6) = mRna_decay_rate*(mRna1); %mRna decay b

b(7) = translation_rate*(mRna0); %enzyme a produce

b(8) = translation_rate*(mRna1); %enzyme b produce

b(9) = Protein_decay*(Arr0); %enzyme a decay

b(10) = Protein_decay*(Arr1); %enzyme b decay

b(11) = ((V*A)*Kcat*(Arr0/(V*A))*(A/(V*A)))/(Km+(A/(V*A))); %B input flux catalyzed by enzyme a

b(12) = ((V*A)*Kcat*(Arr1/(V*A))*(B/(V*A)))/(Km+(B/(V*A))); %c input flux catalyzed by enzyme b

b(13) = Adecay*A;

b(14) = Cdecay*C;

b(15) = InputfluxofA;

end

astr = b(15)+b(14)+b(13)+b(12)+b(11)+b(10)+b(9)+b(8)+b(7)+b(6)+b(5)+b(4)+b(3)+b(2)+b(1);

if~astr, break,end

tau = -1/astr*log(rand);

rt = astr*rand;

totalb = 0;

for k = 1:3

totalb = totalb+b(k);

if(totalb >= rt)

break;

end

end

u = k;

if(u == 1)

B = B + 1;

end

if(u == 2)

C = C + 1;

end

if(u == 3)

mRna0 = mRna0 + 1;

end

if(u == 4)

mRna1 = mRna1 + 1;

end

if(u == 5)

mRna0 = mRna0 - 1;

end

if(u == 6)

mRna1 = mRna1 - 1;

end

if(u == 7)

Arr0 = Arr0 + 1;

end

if(u == 8)

Arr1 = Arr1 + 1;

end

if(u == 9)

Arr0 = Arr0 - 1;

end

if(u == 10)

Arr1 = Arr1 - 1;

end

if(u == 11)

B = B + 1;

end

if(u == 12)

C = C + 1;

end

if(u == 13)

A = A - 1;

end

if(u == 14)

C = C - 1;

end

if(u == 15)

A = A + 1;

end

t=t+tau;

T0(index) = t;

y00(index) = A;

y01(index) = B;

y02(index) = C;

y03(index) = Arr0;

y04(index) = mRna0;

y05(index) = Arr1;

y06(index) = mRna1;

end

figure(1);

plot(T0(1:index),y00(1:index),'-');title('\bf\fontsize{14}A. A'); hold on;

figure(2);

plot(T0(1:index),y01(1:index),'-'); title('\bf\fontsize{14}B. B');hold on;

figure(3);

plot(T0(1:index),y02(1:index),'-'); title('\bf\fontsize{14}C. C');hold on;

figure(4);

plot(T0(1:index),y03(1:index),'-'); title('\bf\fontsize{14}D. Arr0');hold on;

figure(5);

plot(T0(1:index),y04(1:index),'-'); title('\bf\fontsize{14}D. mRna0');hold on;

figure(6);

plot(T0(1:index),y05(1:index),'-'); title('\bf\fontsize{14}D. Arr1');hold on;

figure(7);

plot(T0(1:index),y06(1:index),'-'); title('\bf\fontsize{14}D. mRna1');hold on;
